# Full moon mesencephalon sign: a transient cytotoxic lesion in a hemodialysis patient with newly diagnosed atrial fibrillation and severe stenosis of the right vertebral artery

**DOI:** 10.1007/s10072-024-07926-6

**Published:** 2024-12-11

**Authors:** Celeste Sassi, Sylvia Habermann, Marcelo Alejandro Coria, Albert Grüger, Vasilis Kola, Hans-Michael Schmitt

**Affiliations:** 1 Department of Experimental Neurology, Charité - Universitätsmedizin Berlin, Corporate Member of Freie Universität Berlin, Humboldt- Universität zu Berlin, Berlin Institute of Health, Berlin, Germany; 2 Department of Neurology, Martin Gropius Hospital, Eberswalde, Germany

## Abstract

Brain cytotoxic edema is a neuroradiological sign secondary to variegate diseases ranging from migraine to fulminant Listeria rhombencephalitis. The tempestive identification of its underlying cause is vital for an effective treatment as any delay may be fatal. However, the lack of distinctive imaging biomarkers and the paucity of reports pose a significant challenge in its diagnosis and frequently lead to a misdiagnosis particularly with the more common acute ischemic stroke. Importantly, due to its rarity, mesencephalon midline cytotoxic lesion is likely to remain an underdiagnosed clinical phenomenon, especially if follow up MRI is not performed. Here we report a case of a central, midline, symmetric midbrain cytotoxic edema in a haemodialysis patient with diverse chronic progressive severe cardiovascular risk factors and a newly diagnosed atrial fibrillation. We expand the spectrum of neuroradiological hallmarks associated to terminal renal failure and report a full moon-like mesencephalon midline transient restricted diffusion as a reliable imaging biomarker for the prompt and accurate diagnosis of midbrain cytotoxic edema with the enormous potential of rapidly identifying and effectively treating its causative factors and timely reverse the associated symptomatic.

Dear Editor in Chief,

Brain cytotoxic edema is a neuroradiological hallmark secondary to a wide spectrum of diseases ranging from migraine with aura to fulminant encephalitis [1, 2].

Identifying its underlying cause is critical to guarantee its reversibility as any delay may be fatal [2]. However, the lack of reports, the paucity of distinctive imaging biomarkers pose a major challenge in its diagnosis and frequently lead to a misdiagnosis particularly with the more common acute ischemic stroke [2].

We recently reported a cytotoxic edema of the corpus callosum (CLOCCS) in a patient with COVID-19 Omicron variant [3], supporting a growing body of evidence linking COVID-19 different strain infections to a specific neurotoxic process in the splenium of the corpus callosum [4]. The very peculiar shape and location of this lesion, generally round, oval symmetric in the centre of the corpus callosum splenium has become an emblematic MRI biomarker that proved to be illuminating for the early identification of many other previously unrecognised cases [4]. Here, we report a case of a central, midline, symmetric midbrain cytotoxic edema ventral to the central aqueduct in a haemodialysis patient with multiple severe cardiovascular risk factors.

A 66-year-old patient was admitted for an exacerbation of the known gait disorder, slurred speech, left hemiparesis with ipsilateral central facial palsy since the last dialysis session, 3 days before.

Importantly, he presented multiple acquired cardiovascular risk factors (End-Stage Renal Failure [G5D], decompensated heart failure, hypertensive heart disease, high blood pressure, diabetes mellitus type 2, severe obesity [123,7 Kg, BMI: 54,2 kg/m²] and hyperlipidaemia, which were treated with 3 day a week hemodialysis, candesartan, torsemide, insulin and atorvastatin 80 mg, respectively.

Clinical, radiological and labor findings did not display any evidence for pneumonia or upper respiratory symptoms such as dysgeusia and anosmia. Body temperature was 37.0 °C and oxygen saturation on room air was > 90%. Laboratory test results did not present any acute or exacerbated metabolic disorder but the known chronic end-stage kidney disease (, Krea, 412umol/l), mild increased C-reactive protein level [12.6 mg/l; normal value (NV): < 5 mg/l]. A venous blood gas analysis revealed the absence of metabolic acidosis: pH > 7.35, bicarbonate level < 24mmol/l; normal range, 19-24mmol/L).

The head CT-Scan showed two old bihemispheric lacunar infarcts in the anterior limb of the capsula interna right and in thalamus left and a mild cerebral microangiopathy (Fazekas 1) (Fig. 1 A). Thin-section chest computed tomography did not display cluster-like ground glass opacities, suggestive of COVID-19 Omicron pneumonia [5]. CT-Angiography presented a mild stenosis of the left internal carotid artery (circa 50% by NASCET criteria) and a severe stenosis of the V1 segment of the right vertebral artery, without any features suggestive of vasculitis (Fig. 1 B-C). A mild hypotension (100 mmHg systolic) and a new permanent and normofrequent atrial fibrillation were detected during the Stroke-Unit monitoring.

Brain magnetic resonance imaging (MRI) 2 days after admission (and 6 days after symptom onset) confirmed 2 old ischemic lesions in the right anterior capsula interna and left thalamus and revealed a round and circular and symmetric midline lesion in the central portion of mesencephalon with subtle hyperintensity on fluid attenuated inversion recovery (FLAIR) (Fig. 1D) corresponding to a T2-diffusion-weighted imaging (DWI) with restricted diffusion (Fig. 1E), a low signal intensity on apparent diffusion coefficient (ADC) (Fig. 1F) and a very mild decreased intensity on T1-weighted sequences (Fig. 1G), compatible with the diagnosis of cytotoxic lesion of mesencephalon [1].

**Fig. 1:**
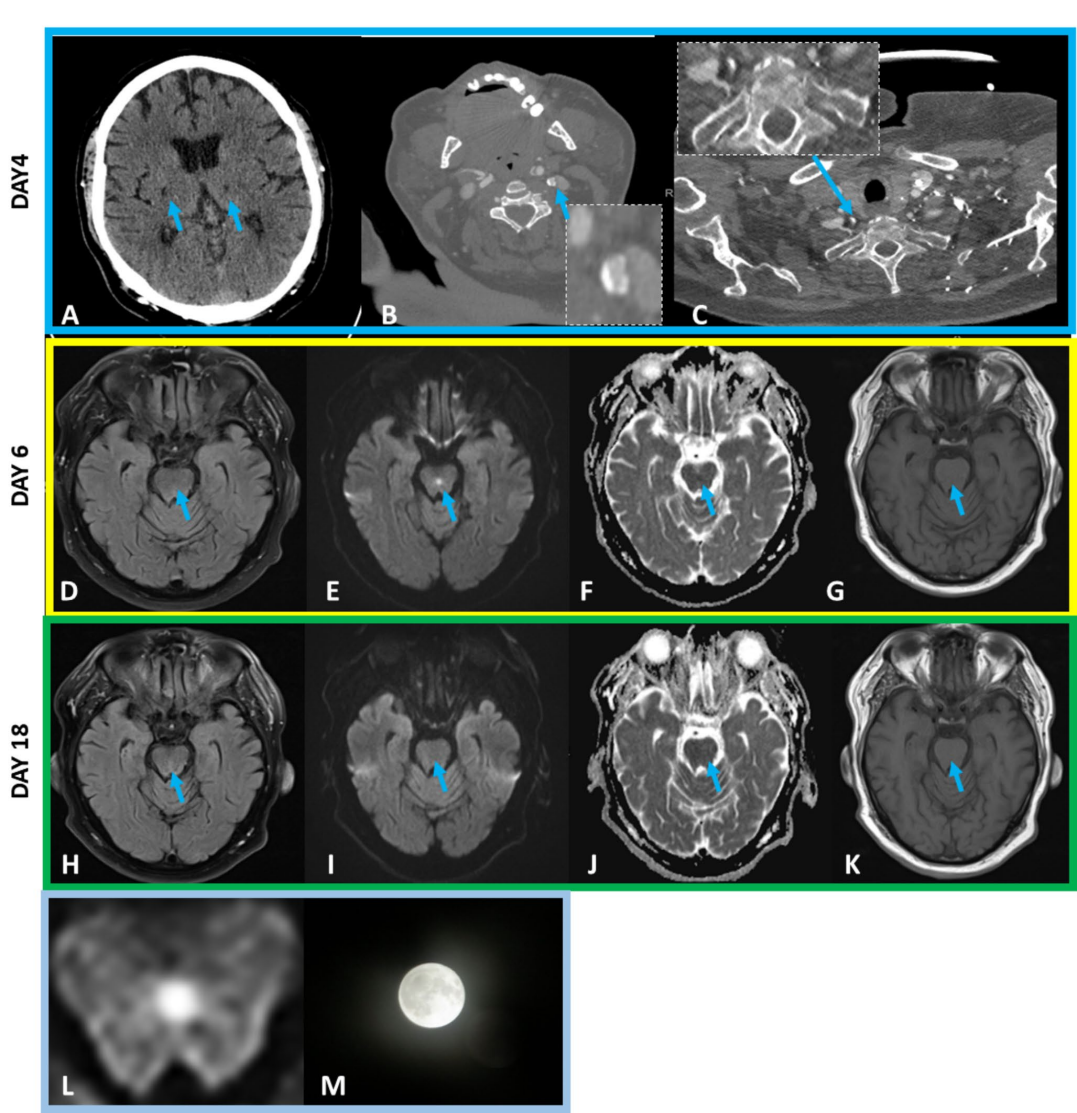
**A-L. Neuroradiological features detected in the described patient**. **A-C Axial Head CT-scan**,** axial CT-Angiography of Head and Neck**,** 4 days after onset of symptoms. A**, Axial-Head-CT-Scan displaying two old lacunar infarcts (anterior limb of the capsula interna right and in thalamus left, blue arrows). **B-C** CT-Angiography of Head and Neck showing a moderate stenosis (ca. 50% by NASCET criteria) of the left internal carotid artery (**B**, blue arrows) and a severe stenosis of the right vertebral artery (V1 Segment) (**C**, blue arrows). **D-G**, axial Brain MRI scans performed 6 days after symptom onset and displaying a central, round, symmetric and bilateral lesion close to Wernekink commissure in the mesencephalon with subtle hyperintensity on fluid attenuated inversion recovery (FLAIR) (**D**) corresponding to a T2-diffusion weighted imaging (DWI) with restricted diffusion (**E**) and low signal intensity on apparent diffusion coefficient (ADC)(**F**) and a very mild hypointense signal on T1-weighted imaging (**G**) (blue arrows).**H-K**, Follow up brain MRI scans performed 18 days post symptom onset presenting a complete resolution of the midbrain lesion in the T2-FLAIR (**H**) as well as in the T2- DWI sequences (**I**), on apparent diffusion coefficient (ADC) (**J**) and on T1-weighted imaging sequences (**K**) (blue arrows), corresponding to the patient clinical remission. **L-M.** T2- Diffusion*-*weighted imaging (DWI) performed 6 days after onset of symptomatic, presenting a midbrain central, symmetric, round lesion with restricted diffusion (**L**), typically resembling the shape of a full moon (**M**)

Laboratory tests excluded possible additional endocrine and metabolic disorders (hypoglycemia, hypo- and hyperthyroidism, hypo- and hyperparathyroidism, hypernatremia and hepatic encephalopathy), infectious and iatrogenic causes of cytotoxic edema were ruled out. A lumbar puncture was refused by the patient.

Given the newly diagnosed atrial fibrillation, the severe stenosis of the right vertebral artery and several cardiovascular risk factors, the patient was initially diagnosed with vertebrobasilar cardioembolic stroke.

Due to the terminal chronic kidney disease an anticoagulation therapy was only possible with unfractionated Heparin 5000 units subcutaneously every 12 h in the non-dialysis days.

During his stay at the hospital his symptoms progressively spontaneously resolved in the next 4 days and he was dismissed with the known gait impairment.

A follow-up MRI 12 days later was performed and given the complete resolution of the midbrain lesion (Fig. 1H-K) an additional MRI scan with Gadolinium was not performed.

Importantly, transitory dysarthria and movement disorders have been already described in dialytic patients with uremic encephalopathy and variable degrees of metabolic acidosis [6]. In these patients the neurological complications associated to chronic renal failure are caused by the accumulation of uremic toxins such as guanidine compounds, which could stimulate the neurotoxic effect of excitatory N-methyl-D aspartate (NMDA) receptors and concomitant inhibition of inhibitory gamma-aminobutyric acid (GABA) receptors. In these cases haemodialysis proved to be therapeutic, fully reversed the initial neurological symptoms and has been associated to a peculiar imaging biomarker: the lentiform fork sign, described as an hyperintense rim delineating the medial and lateral boundaries of the putaminae, resembling a fork [6]. Additionally, a transitory brain hypoperfusion has been frequently reported during intradialytic sessions, progressively leading to leukoaraiosis and cognitive impairment [7]. Moreover, a central pons-mesencephalon T2-DWI hyperintensity has been already detected among a end-stage renal disease patient on haemodialysis experiencing acute neurologic complications [8]. Finally, similar central mesencephalon lesions have been described in Listeria rombehcephalitis, whose initial misdiagnosis with vertebrobasilar ischemic stroke led only to a post-mortem diagnosis [2].

By contrast, isolated mesencephalic ischemic lesions have been mostly described in the Wernekink Syndrom, a very rare disorder, as to date only 17 cases have been reported [9, 10], characterized by a clinical spectrum including cerebellar dysarthria, bilateral ataxia, oculomotor dysfunction, internuclear ophthalmoplegia, and delayed-onset palatal myoclonus [11]. An acute infarct of the Wernekink commissure appears as a mostly unilateral round, oval, heart, or V-shaped hyperintensity on DWI in the caudal paramedian midbrain, ventral to the cerebral aqueduct [9].

In this patient, we hypothesized that the cytotoxic lesion may have been caused by a pre-existing arterial hypotension which may have further worsened the intradialytic transient brain hypoperfusion and may have delayed the clearance of neurotoxic substances [12].

Importantly, although brain cytotoxic edema imaging biomarkers share distinctive features: they are symmetric, regular, round, bilateral, central and, interestingly, mostly in territories of vertebrobasilar pertinence (thalamus, corpus callosum splenium, occipital cortex) [3, 6], due to its rarity, brainstem and particularly mesencephalon midline cytotoxic lesion is likely to remain an underrecognized and underdiagnosed clinical phenomenon, especially if control MRI is not performed.

Here, we expand the neuroradiological signs associated to end-stage renal failure and report a full moon-like mesencephalon midline transient restricted diffusion (Fig. 1 E, L, M) as a reliable imaging biomarker, enabling the tempestive and accurate diagnosis of midbrain cytotoxic edema with the enormous potential of rapidly identifying and effectively treating the underlying cause and reversing the symptoms.

Our case-report should foster a validation in large cohorts of end-stage haemodialysis patients.
